# Nitric oxide-releasing porous silicon nanoparticles

**DOI:** 10.1186/1556-276X-9-333

**Published:** 2014-07-04

**Authors:** Morteza Hasanzadeh Kafshgari, Alex Cavallaro, Bahman Delalat, Frances J Harding, Steven JP McInnes, Ermei Mäkilä, Jarno Salonen, Krasimir Vasilev, Nicolas H Voelcker

**Affiliations:** 1 ARC Centre of Excellence in Convergent Bio-Nano Science and Technology, Mawson Institute, University of South Australia, GPO Box 2471 Adelaide, SA 5001, Australia; 2 Department of Physics and Astronomy, University of Turku, Turku FI-20014, Finland

**Keywords:** Porous silicon nanoparticles, Nitric oxide, Antibacterial

## Abstract

In this study, the ability of porous silicon nanoparticles (PSi NPs) to entrap and deliver nitric oxide (NO) as an effective antibacterial agent is tested against different Gram-positive and Gram-negative bacteria. NO was entrapped inside PSi NPs functionalized by means of the thermal hydrocarbonization (THC) process. Subsequent reduction of nitrite in the presence of d-glucose led to the production of large NO payloads without reducing the biocompatibility of the PSi NPs with mammalian cells. The resulting PSi NPs demonstrated sustained release of NO and showed remarkable antibacterial efficiency and anti-biofilm-forming properties. These results will set the stage to develop antimicrobial nanoparticle formulations for applications in chronic wound treatment.

## Background

Wound contamination by bacteria or other microorganisms may cause a delay in or a deterioration of the healing process [1,2]. Although bacteria are present in most wounds, the body’s immune defense is generally efficient in overcoming this contamination and supporting successful healing. However, in some cases, such as diabetic, immunocompromised or elderly patients, the immune system requires assistance [3-6]. Typical treatments for infection in these cases include antibiotics, which can be applied directly to the wound or taken orally. In cases of severe infection, intravenous administration is required to rapidly achieve dosages sufficient to clear the bacterial load [7,8]. Recently, concerns have arisen over the increased prevalence of antibiotic-resistant bacteria such as methicillin-resistant *Staphylococcus aureus* (MRSA), which is promoted by injudicious antibiotic use [3,9]. Serious and sometimes fatal cases of antibiotic-resistant infections have occurred in hospitals and community settings [10], and this is developing into an important public health problem [8].

Recently, new antibacterial therapeutics based on nanomaterials have emerged for the treatment of infected wounds [11-14]. For example, mesoporous silica has been used as a nanocarrier to deliver antibacterial agents lysozyme and 1-alkylquinolinium bromide ionic liquids in a controlled manner [15,16]. However, the further development of antibiotic delivering nanoparticles (NPs) has been hampered by increasing bacterial resistance to conventional antibiotic candidates for the active agent [3]. In the early 1990s, nitric oxide (NO) was considered as an alternative antibiotic strategy for a wide range of Gram-positive and Gram-negative bacteria [17,18]. NO is produced by various cells resident in the skin as one of the natural defenses of the immune system and should therefore prove to be effective against pathogen invasion while being tolerated by human skin [19]. The mechanism of NO-mediated bactericidal actions is reasonably well understood [19,20]. A major factor appears to be membrane destruction via lipid peroxidation [9,17].

In order to harness the antibacterial power of NO, however, this molecule must be loaded and trapped in a suitable carrier. NO-loaded silica nanocarriers have been synthesized using diazeniumdiolate NO donors [9]. The NO loading capacity was directly influenced by NP size [21]. These NPs showed antibacterial efficacy in a time- and concentration-dependent manner [9,21] and reduced biofilms composed of Gram-positive and Gram-negative bacteria (≥5 and 2 log reduction, respectively) [22]. In an alternative approach, Friedman and co-workers synthesized NO-loaded silica nanocarriers using glucose for the thermal reduction of nitrite to NO [23]. The sustained release of NO from the silica NPs resulted in antimicrobial and wound-healing properties against cutaneous MRSA and *Acinetobacter baumannii *[4,23].

Porous silicon (PSi) is a high surface area, high porosity, biocompatible, and bioresorbable form of silicon widely employed in biomedical applications, including as NPs [24-28]. The use of PSi NPs avoids the issues of toxicity associated with silica-derived nanocarriers; further, NP porosity can be easily tuned by manipulation of current density [29,30]. Thermally hydrocarbonized porous silicon (THCPSi) NPs have remarkable stability in physiological environments and also show low cytotoxicity *in vivo *[25]. THCPSi elicits little inflammatory response [25,28]. Small molecular drugs and peptides have been successfully loaded into and released from THCPSi NPs, with some promising results in the areas of drug delivery and multimodal bioimaging [24]. Due to these promising properties, we have chosen THCPSi NPs as a nanocarrier for NO and have explored the antibacterial efficacy of NO-loaded NPs towards planctonic *Escherichia coli*, *Pseudomonas aeruginosa*, and *Staphylococcus aureus* and a *Staphylococcus epidermidis* biofilm. All of these pathogens can cause primary skin and soft tissue infection [8,31,32]. We also investigated whether the same NPs would be cytotoxic to fibroblast cells.

## Methods

### Chemicals and materials

Silicon wafers (boron doped, p^+^ type, 0.01 to 0.02 Ω cm) were obtained from Siegert Wafer GmbH (Aachen, Germany). Ethanol (EtOH, 99.6 vol.%) was obtained from Altia Plc. (Porkkalankatu, Finland), and hydrofluoric acid (HF, 38%) from Merck GmbH (Darmstadt, Germany). Sulfuric acid, sodium nitrite, Griess reagent, 4-amino-5-methylamino-2′,7′-difluorofluorescein (DAF-FM), d-glucose, potassium hydroxide, and phosphate-buffered saline (PBS) tablets were purchased from Sigma-Aldrich (St. Louis, MO, USA). Tryptic soy broth (TSB; soybean-casein digest) and nutrient agar were purchased from Thermo-Scientific (Waltham, MA, USA). *E. coli* (ATCC #25922), *P. aeruginosa* (ATCC #27853), *S. epidermidis* (ATCC #35984), and *S. aureus* (ATCC #29213) were obtained from the American Type Culture Collection (Manassas, VA, USA). For mammalian cell culture, the following reagents were used as received: 0.01 M PBS pH 7.4 (Sigma-Aldrich), DMEM medium, fetal bovine serum (FBS), l-glutamine, penicillin, streptomycin, amphotericin B (all purchased from Life Technologies, Carlsbad, CA, USA), propidium iodide (PI; Sigma-Aldrich), fluorescein diacetate (FDA; Sigma-Aldrich), lactate dehydrogenase (LDH) cytotoxicity assay kit II (Abcam, Cambridge, UK), and trypsin (0.05%, EDTA 0.53 mM, Life Technologies). Cell culture media were prepared using ultrapurified water supplied by a Milli-Q system (Millipore Co., Billerica, MA, USA). NIH/3T3 mouse embryonic fibroblasts (ATCC #CRL-1658) from the American Type Culture Collection were used in these experiments.

### Fabrication of THCPSi NPs

THCPSi NPs were fabricated according to the previously reported procedure [25] from p^+^ type (0.01 to 0.02 Ω cm) silicon wafers by periodically etching at 50 mA/cm^2^ (2.2-s period) and 200 mA/cm^2^ (0.35-s period) in an aqueous 1:1 HF(38%)/EtOH electrolyte for a total etching time of 20 min. Subsequently, the THCPSi films were detached from the substrate by abruptly increasing the current density to electropolishing conditions (250 mA/cm^2^, 3-s period). The detached multilayer films were then thermally hydrocarbonized under N_2_/acetylene (1:1, volume) flow at 500°C for 15 min and then cooled down to room temperature under a stream of N_2_ gas. The THCPSi membranes (1.3 g) were converted to NPs using wet ball milling (ZrO_2_ grinding jar, Pulverisette 7, Fritsch GmbH, Idar-Oberstein, Germany) in 1 decene (18 mL) overnight. A size separation was performed by centrifugation (1,500 RCF, 5 min) in order to achieve a narrow particle size distribution.

### Preparation of NO/THCPSi NPs

Sodium nitrite (10 mM) dissolved in 50 mM PBS (pH 7.4) was mixed with glucose 50 mg/mL. The THCPSi NPs were then added to this buffer solution at different concentrations (ranging from 0.05 to 0.2 mg/mL). Subsequently, the suspension was sonicated for 5 min to ensure particle dispersion and then stirred for 2 h. Upon NO incorporation, the THCPSi NPs were centrifuged at 8,000 RCF for 10 min for collection. Finally, after removing the supernatant, the THCPSi NP pellet was dried by heating at 65°C overnight. The drying temperature was held at 70°C to avoid glucose caramelization [23,33,34]. An alternative drying procedure, overnight lyophilization (FD1 freeze dryer, Dynavac Co., MA, USA), was also assessed, as described in the text [23].

Glucose/THCPSi NPs and sodium nitrite/THCPSi NPs were also prepared following the same procedure as for the NO/THCPSi NPs but omitting either sodium nitrite or d-glucose during NP loading, respectively. All prepared NPs were kept at ambient conditions and were dispersed via sonication for 5 min in PBS before use.

### Pore structure analysis

The pore volume, average pore diameter, and specific surface area of the THCPSi NPs were calculated from nitrogen sorption measurements on a TriStar 3000 porosimeter (Micromeritics Inc., Norcross, GA, USA).

### Scanning electron microscopy

Morphological studies of THCPSi NPs were carried out by means of scanning electron microscopy (SEM) on a Quanta™ 450 FEG instrument (Hillsboro, OR, USA) by collecting secondary electrons at 30-kV beam energy under high vacuum of 6 × 10^-4^ Pa. Energy-dispersive X-ray spectroscopy (EDX) measurements were performed using a Link 300 ISIS instrument from Oxford Instruments (detector Si(Li), 30-kV beam energy, resolution 60 eV; Abingdon, Oxfordshire, UK). The samples were prepared by fixing the NPs to the microscope holder, using a conducting carbon strip. In order to conduct SEM and EDX analysis of NO/THCPSi NPs treated and untreated with *E. coli*, colonies at the desired growth stage were fixed by formaldehyde (4 *v*/*v*%) for 2 h on round graphite disks. After rinsing twice with PBS, the disks were attached on a SEM holder and were observed by using the Quanta™ 450 FEG SEM and the Link 300 ISIS EDX (Oxford Instruments).

### Dynamic light scattering

The mean particle size and size distribution of NPs were determined by dynamic light scattering (DLS; Zetasizer Nano ZS, Malvern Instruments, Malvern, UK). The analysis was carried out at a temperature of 25°C using NPs dispersed in ultrapurified water. Every sample measurement was repeated 15 times.

### Infrared spectroscopy

Diffuse reflectance infrared Fourier transform (DRIFT) spectra were acquired using a Thermo Nicolet Avatar 370MCT (Thermo Electron Corporation, Waltham, MA, USA) instrument. A smart diffuse reflectance accessory was used for all samples embedded within KBr pellets. The spectra were recorded and analyzed using OMNIC version 7.3 software (Thermo Electron Corp., Waltham, MA, USA). For each spectrum, 128 scans were averaged in the range of 4,000 to 800 cm^-1^ with a resolution of 4 cm^-1^. In addition, dipole moments of the chemicals were calculated using the Millsian 2.1 Beta (Millsian, Inc., Cranbury, NJ, USA). Background spectra were blanked using a suitable clean silicon wafer. All spectra were run in dry air to remove noise from CO_2_ and water vapor.

### Generation of NO

A calibration curve for NO was obtained by preparing a saturated solution of NO as described previously by Mesároš et al. [35]. Briefly, 10 mL of PBS (pH 7.4) was degassed using an Ar purge for 60 min. Subsequently, NO was generated by adding 20 mL of 6 M sulfuric acid slowly to 2 g of sodium nitrite in a twin-neck round-bottom flask, which was connected via rubber tubing to a Büchner flask containing KOH solution (to remove NO degradation products, 10% *v*/*v*). The Büchner flask was then connected to the flask containing degassed PBS. The NO gas produced was bubbled through the degassed PBS (held at 4°C) for 30 min to produce a saturated NO solution. The solubility of NO in PBS at atmospheric pressure is 1.75 ± 0.02 mM [35-37]. Using Griess reagent [13], our solution was found to have a concentration of 1.87 mM at 37°C.

### Colorimetric assay of nitrite

The presence of nitrite compounds can be detected by the Griess reaction, which results in the formation of a characteristic red pink color. Nitrites react with sulfanilic acid to form a diazonium salt, which then reacts with *N*-alpha-naphthyl-ethylenediamine to form a pink azo dye [38,39]. A calibration curve was prepared using dilutions of sodium nitrite between 0.43 and 65 μM in PBS (pH 7.4, temperature 37°C) mixed with equal volumes of the prepared Griess reagent according to the manufacturer’s instructions. The absorbance of the solutions at 540 nm was measured on a HP8453 PDA UV/VIS spectrophotometer (Agilent, Santa Clara, CA, USA).

### Fluorimetric determination of NO

To detect the release of NO from PSi NP, the DAF-FM assay was used. DAF-FM is non-fluorescent until it reacts with NO to form a fluorescent benzotrizole. DAF-FM possesses good specificity, sensitivity (approximately 3 nM) and is simple to use [23,36]. It does not react with the other nitrogen oxides (i.e., NO_2_^-^ and NO_3_^-^) and reactive oxygen species (i.e., O_2_^-^ and H_2_O_2_) [23].

Fluorescence spectra for all samples were acquired using a LS 55 spectrofluorometer (PerkinElmer, Waltham, MA, USA) with slit widths set at 2.5 nm for both excitation and emission; the photomultiplier voltage was set to 775 V, and a wavelength of 495 nm was used for excitation and 515 nm for emission. In order to prepare an approximate 1 mM stock DAF-FM solution, 1 mg of DAF-FM was dissolved in 250 μL DMSO and then the stock solution (10 μL) was mixed with 90 μL PBS (pH 7.4). Fluorescence was expressed as arbitrary fluorescence units and was measured at the same instrument settings in all experiments.

For the fluorescence-based measurements of NO concentration, a calibration curve was prepared using dilutions of saturated NO solution in PBS between 0.00 and 1.87 mM in PBS (pH 7.4, 37°C). Fresh DAF-FM stock solution was added to the PBS and immediately mixed in an Eppendorf tube in the darkness using a shaker for 2 min and then transferred into a quartz cuvette with a stopper, and the fluorescence was measured after a 5-min incubation.

### Nitric oxide release from NO/THCPSi NPs

The prepared NO/THCPSi NPs (0.1 mg/mL) were added to PBS (1 mL), sonicated, and mixed using a test tube shaker. After incubation at 37°C for the sampling interval times specified in the text, the NPs were centrifuged at 12,000 RCF for 5 min and then the supernatant containing the released NO from the NPs was separated and pre-incubated with 2 μL DAF-FM solution (approximately 1 mM) for 2 min at room temperature in the darkness on a test tube shaker (approximately 0.1 RCF). The supernatant containing NO and DAF-FM was subsequently transferred into a cuvette, and fluorescence intensities were measured as described above. The amount of the released NO was calculated using the fluorimetric DAF-FM calibration curve.

### Determination of antimicrobial activity

*P. aeruginosa*, *E. coli*, and *S. aureus* were cultured overnight at 37°C in TSB and diluted to a concentration of 10^8^ colony-forming units per milliliter (CFU/mL) based on turbidity (OD_600_) and further diluted to 10^4^ CFU/mL and 1 mL treated with different concentrations of NO/THCPSi NPs or glucose/THCPSi NPs (control). As a further control, NO/THCPSi NPs (0.1 mg/mL) were added to 0.5 mL of PBS, sonicated for 5 min and then incubated for 2 h to remove NO, centrifuged (12,000 RCF for 5 min), and NO-depleted NO/THCPSi NPs dried at 65°C overnight. Bacteria not treated with NPs were used as negative controls in each experiment.

The NP samples were incubated for 2 h, 4 h (*S. aureus*; 0.05, 0.1, or 0.2 mg/mL concentration of NPs), and 24 h (*P. aeruginosa*, *E. coli*, and *S. aureus*; 0.1 mg/mL concentration of NPs) at 37°C. *S. aureus* were then serially diluted and spread-plated on nutrient agar. Bacterial viability was assessed by counting the number of colonies formed on the agar plate. The colony count was normalized by considering the untreated colony (negative) as 100% of bacteria viability. The viability of *E. coli* and *P. aeruginosa* after 24 h was determined by turbidity measurements (OD_600nm_), taking into account background caused by the NPs themselves.

### Effect of NO/THCPSi NPs on established biofilms

The reduction in total viable cells recovered from established *S. epidermidis* biofilms treated with NO/THCPSi NPs was compared to the control biofilms of the same species not treated with the NPs. Glass microscope slides were cut into pieces with surface areas of 24 mm^2^. The glass pieces were cleaned with 70% ethanol and dried. *S. epidermidis* was cultured at 37°C in TSB overnight and diluted to 10^6^ CFU/mL. The 10^6^ CFU/mL microbial suspension was then added to each tube containing the glass slide pieces. The vials containing bacteria, broth, and glass slide pieces were placed in a 37°C incubator for biofilm formation. After 24 h, the glass slide pieces were removed from the nutrient broth, rinsed twice in sterile PBS, and individually transferred into new Eppendorf tubes containing a fresh suspension 1 mL of 0.1 mg/mL NO/THCPSi NPs and THCPSi NPs (control) in PBS and returned to the 37°C incubator. After 24 h, the tubes containing glass slide pieces were sonicated in a 125-W ultrasonic cleaner for 5 min to remove the biofilm-forming cells from the slide. The resulting bacterial suspension was subjected to serial tenfold dilutions, and 100 μL of appropriate dilutions was plated onto agar plates, which were then incubated at 37°C overnight. The total number of colonies that grew on each plate was counted, and the number of viable biofilm bacteria removed from each slide was determined.

### Mammalian cell viability assay

The cytotoxicity of the NO/THCPSi NPs was evaluated using NIH/3T3 fibroblast cells. The cells were maintained in DMEM supplemented with 10% FBS and 2 mM l-glutamine, 100 U/mL penicillin, 100 μg/mL streptomycin, and incubated at 37°C with 5% CO_2_.

All mentioned procedures for the preparation of NO/THCPSi NPs and glucose/THCPSi NPs were done under sterile conditions within a biological safety cabinet (Bio-cabinet, Aura 2000, Microprocessor Automatic Control, Firenze, Italy).

The NIH/3T3 cells were trypsinized and then seeded into polystyrene 96-well plates (Nalge Nunc International, Penfield, NY, USA) at a density of 3 × 10^4^ cells/mL and then after 24 h, the cultured cells were incubated with NO/THCPSi NPs, glucose/THCPSi NPs, and THCPSi NPs at four different concentrations from 0.05 to 0.2 mg/mL for 48 h.

After the incubation period, the culture medium was separated from the cultured cells and subjected to a LDH assay that was carried out following the manufacturer’s instructions. Moreover, a FDA-PI assay was performed on the cultured cells remaining in the wells. The cells were incubated with fresh medium before adding final concentrations of 15 μg/mL FDA and 5 μM PI for 3 min at 37°C to count the live and dead cells, respectively, using a fluorescence microscope (Eclipse, Ti-S, Nikon, Tokyo, Japan) and determine the percentage of live cells. All experiments were repeated at least three times.

### Statistics

For the NO release tests and bactericidal assays conducted in the related media, *n* = 3 and the data are expressed as mean values ± standard deviation. Statistical significance between populations was determined by one-way ANOVA followed by Tukey’s multiple comparison *post hoc* analysis (GraphPad Prism® software). Data from both the FDA-PI and LDH cytotoxicity assays are presented as mean values ± standard error of the mean.

## Results and discussion

### Characterization of NO/THCPSi NPs

THCPSi NPs were prepared using PSi films fabricated by pulsed electrochemical etching of silicon wafers with (HF; 38%) and ethanol. The preparation and physicochemical characterization of the THCPSi NPs have been described in detail elsewhere [24-26]. Briefly, THCPSi NPs were prepared by using wet ball milling of the multilayer THCPSi films. The described method produced PSi NPs with an average pore diameter of 9.0 nm, a specific surface area of 202 m^2^/g, and a pore volume of 0.51 cm^3^/g. The NPs were NO-loaded via glucose-mediated reduction of nitrite during incubation with THCPSi NPs. Two methods of thermal reduction were assessed: one using lyophilization and one employing heat [23]. The hydrodynamic diameter of the THCPSi NPs and NO/THCPSi NPs was found to be 137 and 142 nm, respectively, according to dynamic light scattering measurements (Additional file 1: Figure S1). The measured zeta (ζ)-potentials of the THCPSi and NO/THCPSi NPs were -30 and -42 mV, respectively.

**Figure 1:**
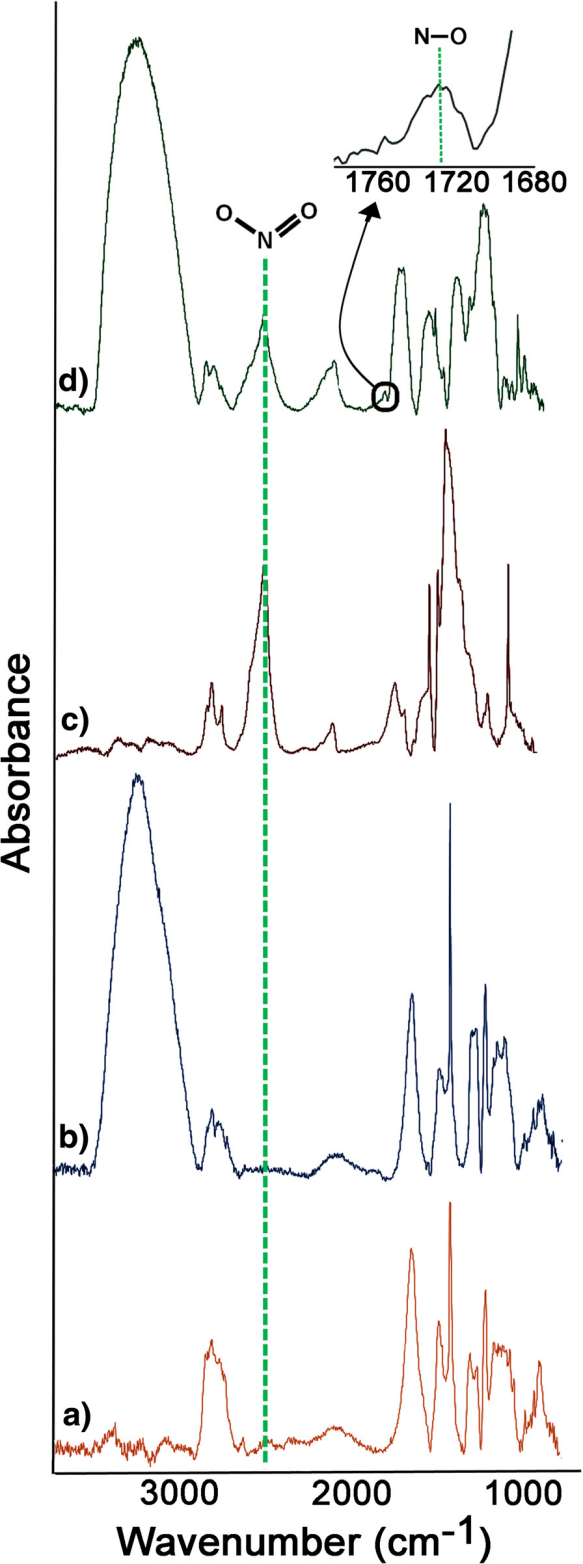
**DRIFT absorbance spectra for PSi NPs. (a)** THCPSi NPs, **(b)** glucose/THCPSi NPs, **(c)** sodium nitrite/THCPSi NPs, and **(d)** NO/THCPSi NPs.

DRIFT spectroscopy was used to chemically characterize PSi NPs. In order to scrutinize the nitrite reduction reaction used to prepare the NO/THCPSi NPs, DRIFT spectra of the prepared THCPSi NPs (control a), glucose/THCPSi NPs (control b), sodium nitrite/THCPSi NPs (control c), and NO/THCPSi NPs were obtained (see Figure 1). The DRIFT spectra obtained from all PSi NPs showed a common set of bands, such as C-H vibration (2,856 cm^-1^), related to the thermal hydrocarbonization [40]. The NO/THCPSi NPs spectrum presented a N-O stretching vibration (dipole moment 0.4344 Debye) at 1,720 cm^-1^, indicating entrapment of NO within the NPs [41]. Moreover, in the spectra of the NO/THCPSi NPs and sodium nitrite/THCPSi NPs, an intense combination band corresponding to O-N = O around 2,670 cm^-1^ was observed [42]. The band related to the O-N = O bending vibration (dipole moment 3.8752 Debye) in the NO/THCPSi NPs is likely to be the result of unreduced sodium nitrite remaining in the NPs. In addition, the presence of the O-H stretching vibrations for NO/THCPSi NPs and glucose/THCPSi NPs indicates the presence of glucose on the NO/THCPSi NPs. A 35% decrease in nitrite band intensity compared to sodium nitrite/THCPSi NPs (normalized between spectra based on C-H vibration at 2,856 cm^-1^) is evidence of the reduction reaction of nitrite during preparation of NO/THCPSi NPs.

### NO release from NO/THCPSi NPs

Sugar-mediated thermal reduction of nitrite-loaded THCPSi NPs produces and entraps NO inside of THCPSi NPs [18,33]. NO formation is the consequence of chemical acidification and redox conversion. Upon drying, d-glucose is oxidized, and correspondingly, nitrite within the pore structure is converted to NO [43]. The dried glucose layer also assists in trapping inside the pores. The entrapped NO is retained within the pores of the NPs until exposed to moisture [18,23].

**Figure 2:**
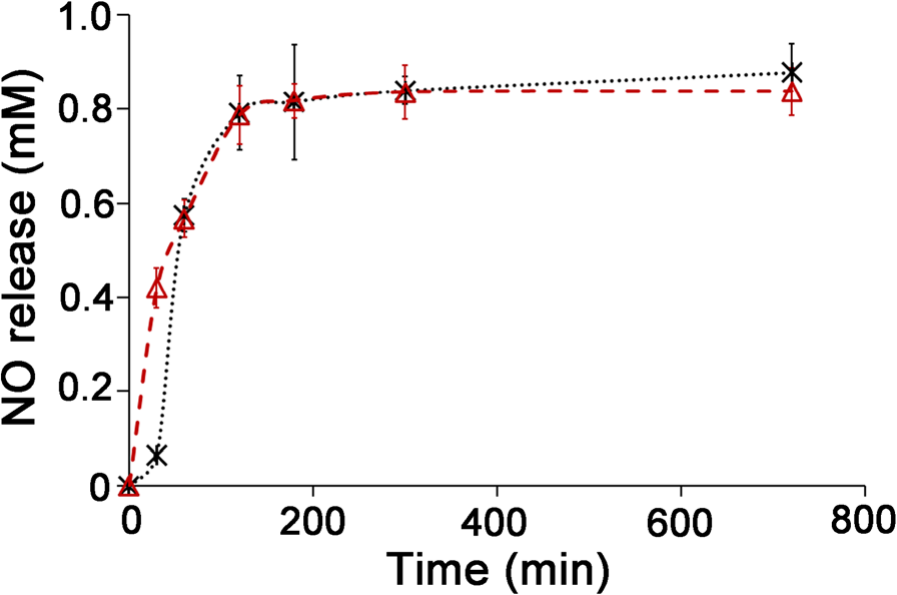
**NO release from NO/THCPSi NPs as a function of time.** NO/THCPSi NPs prepared using the heating protocol (black cross-lines) and the lyophilization protocol (red empty triangles). *n* = 3; mean ± standard deviation shown.

The cumulative release of NO from NO/THCPSi NPs was assessed in PBS (pH 7.4) at 37°C by monitoring conversion of DAF-FM to fluorescein via fluorimetry. DAF-FM conversion requires NO and does not occur in the presence of other reactive oxygen/nitrogen species. The results are shown in Figure 2. NO/THCPSi NPs prepared by both heating and lyophilization protocols were tested. Release of NO from NO/THCPSi NPs occurred predominately in the first 2 h of the monitoring period. Although NPs created by either methods displayed the same maximal release of NO into the PBS medium after 2-h incubation, release profiles obtained using NPs prepared using the lyophilization protocol showed an initial burst release phase (within the first 30 min). In contrast, glucose/THCPSi NPs, sodium nitrite/THCPSi NPs, PBS, and sodium nitrite solution controls showed no NO release (Additional file 1: Figure S2), demonstrating that the NO release indeed only occurs upon nitrite reduction. In reports describing other NO-releasing mesoporous nanocarriers [9,23], only a short period of continuous release is noted, suggesting that the NO/THCPSi NPs described here possess a higher capacity for sustained release of NO.

### Antibacterial efficacy of NO/THCPSi NPs

**Figure 3:**
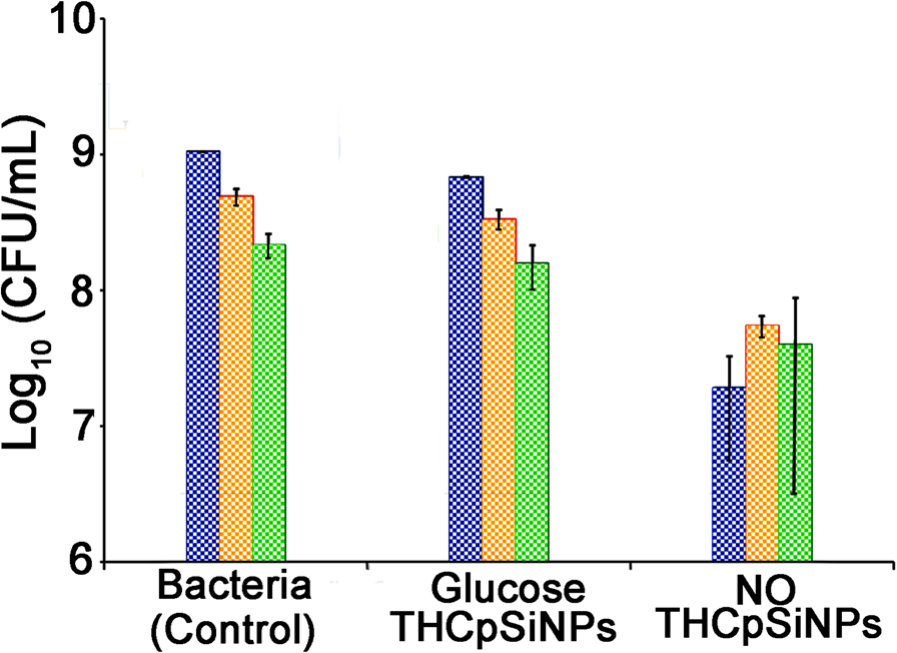
**Inhibitory effect of NO/THCPSi NPs (0.1 mg/mL) on bacterial cultures.***E. coli* (blue bars), *S. aureus* (yellow bars), and *P. aeruginosa* (green bars) after 24 h of incubation in TSB medium (37°C, initial bacteria density 10^4^ CFU/mL; *n* = 3; mean ± standard deviation shown).

Wound contamination by pathogens such as *P. aeruginosa*, *S. aureus*, and *E. coli* is responsible for a significant morbidity load, particularly in burns and immunocompromised patients [8,31,32]. Initial tests of the antibacterial activity of NO/THCPSi NPs (fabricated by the heating method) were performed against planctonic *P. aeruginosa*, *E. coli*, and *S. aureus* (10^4^ CFU/mL for all) treated with 0.1 mg/mL of NPs for 24 h. Compared to the controls (the bacteria cultured without NPs and bacteria treated with glucose/THCPSi NPs), the NO/THCPSi NPs showed significant growth inhibition against all three bacteria species tested (see Figure 3). After the 24-h incubation with 0.1 mg/mL of NO/THCPSi NPs, the bacterial counts of *P. aeruginosa*, *S. aureus*, and *E. coli* cultures were reduced approximately 1 log in comparison with bacteria cultured in the absence of NPs.

**Figure 4:**
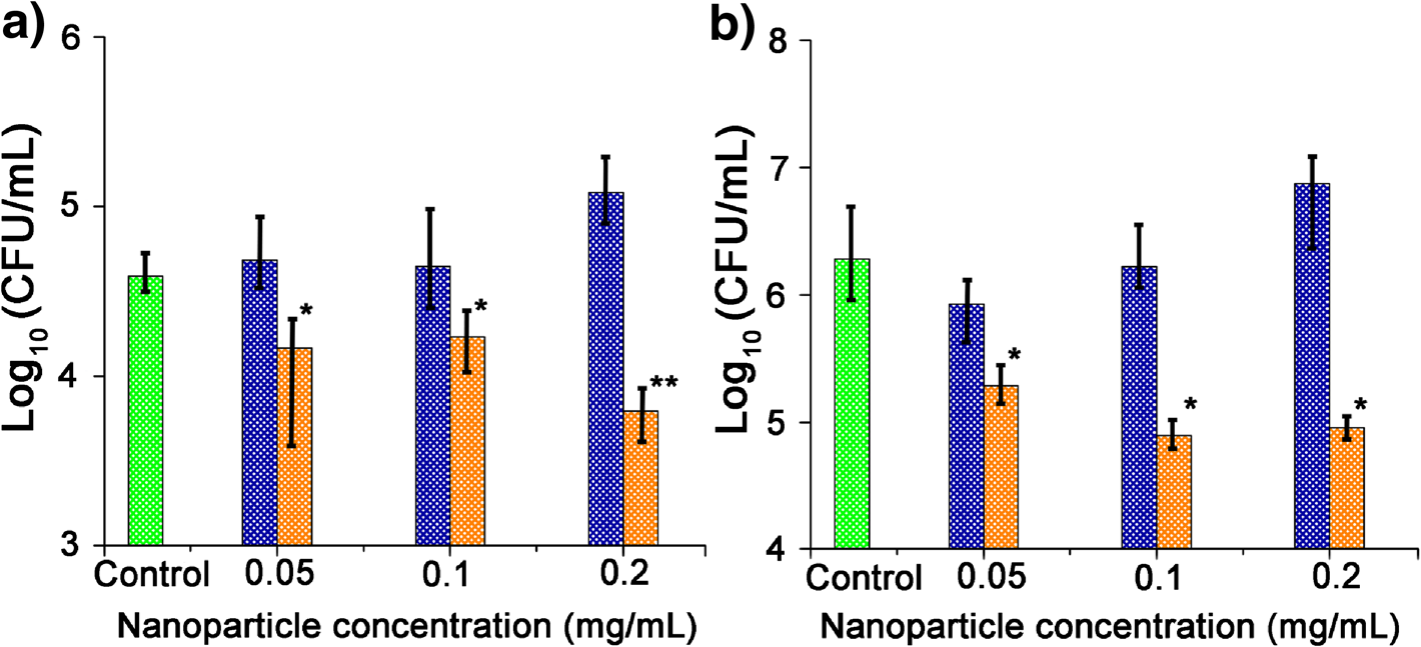
**Time-based inhibition of *S. aureus *by NO/THCPSi NPs.***S. aureus* was treated with glucose/THCPSi NPs (blue columns) and NO/THCPSi NPs (orange columns) at different NP concentrations after **(a)** 2 h and **(b)** 4 h (initial bacteria density 10^4^ CFU/mL). Statistically significant inhibition as compared with control (*P < 0.05, **P < 0.01; *n* = 3; mean ± standard deviation shown).

Further experiments showed that growth inhibition by NO/THCPSi NPs against planktonic *S. aureus* was evident as early as 2 to 4 h after NP treatment (Figure 4). After 2 h, the bacterial counts were reduced by 0.52 log compared to the control (bacteria only), and after 4 h, a further reduction occurred (1.04 log). In contrast, glucose/THCPSi NPs supported *S. aureus* proliferation at the same incubation times. Growth inhibition of *S. aureus* was sensitive to the dose of NO/THCPSi NPs applied (Figure 4). When higher concentrations of NO/THCPSi NPs were applied, the *S. aureus* bacterial load decreased by 1.3 log. It should be noted that a by-product of increasing NP concentration is glucose supplementation, which may be reflected by the increase in bacterial density in cultures treated with glucose/THCPSi NPs. Cultures treated with NO/THCPSi NPs, however, showed no such upward trend in bacterial growth rate, suggesting that the release of NO was able to counter any influence wrought by additional glucose provided by NO/THCPSi NPs. Therefore, these results indicate that the NO released form the NO/THCPSi NPs is an effective antimicrobial agent against medically relevant Gram-positive and Gram-negative bacteria.

**Figure 5:**
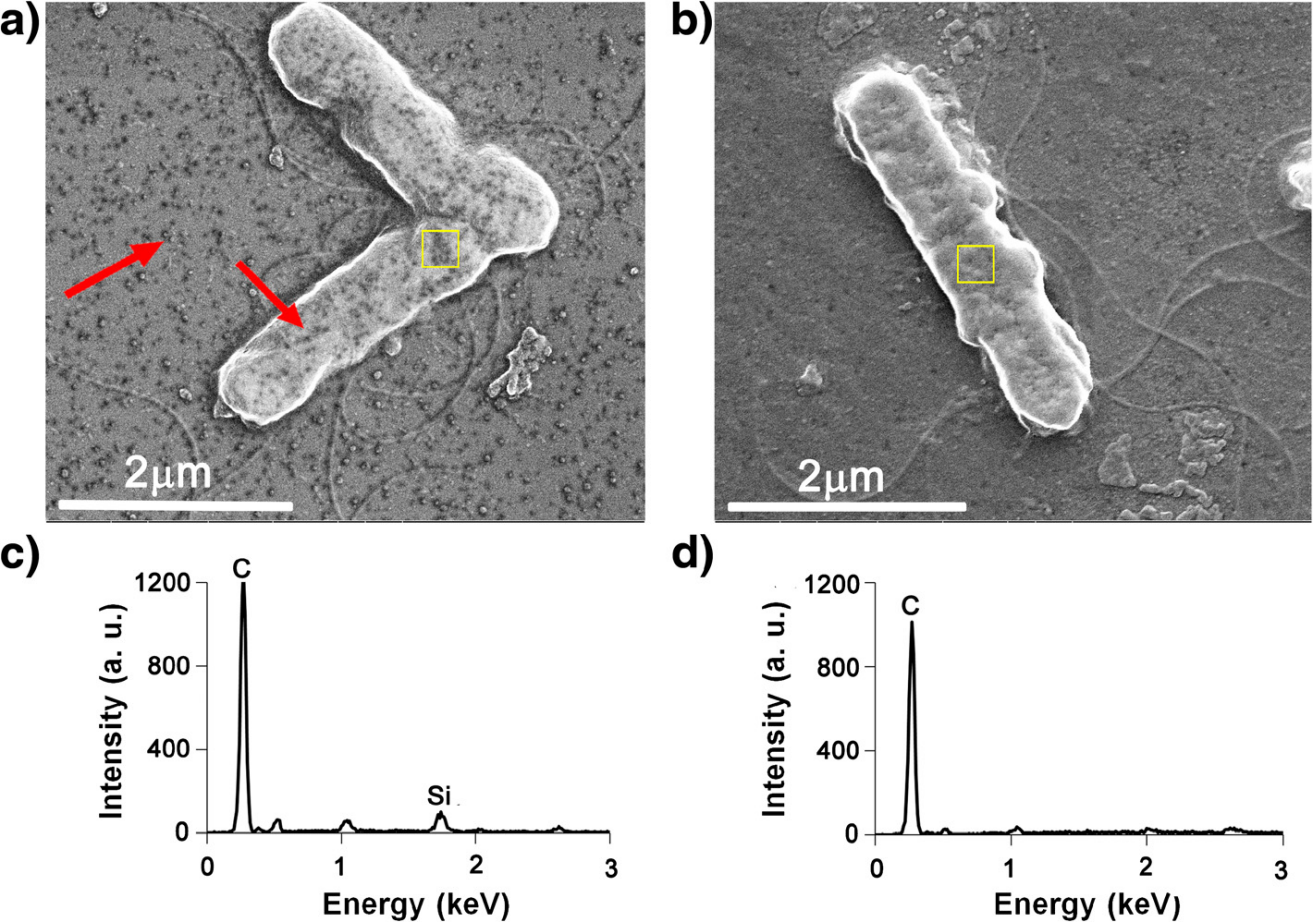
**SEM images and EDX spectra of NO/THCPSi NP-treated *E. coli*. (a)** SEM image of NO/THCPSi NP-treated *E. coli*, **(b)** SEM image of the *E. coli* only, **(c)** EDX spectrum of NO/THCPSi NP-treated *E. coli*, and **(d)** EDX spectrum of untreated *E. coli* as a control. EDX analysis performed on bacterial surface (yellow overlay). NPs on the bacterial surface and settled on the background are indicated by red arrows.

Figure 5 shows the SEM images and EDX spectra of *E. coli* treated with NO/THCPSi NPs compared with an untreated control. Single NPs and NP aggregates were evident in the SEM images on the bacteria and on the background surface. The presence of the NO/THCPSi NPs on the surface of the cell membrane of the *E. coli* was confirmed by the EDX results, which showed a peak characteristic for Si (Figure 5c).

### Anti-biofilm efficacy of NO/THCPSi NPs

*S. epidermidis* biofilms were exposed to the NO/THCPSi NPs at a concentration of 0.1 mg/mL and showed a 0.28 log (47%) reduction in total viable cells compared to the control samples (bacteria only). THCPSi NPs that were not loaded with NO applied at the same concentration produced a negligible reduction in the biofilm density, indicating that the NO released from the prepared NO/THCPSi NPs was the primary cause of any antimicrobial action. In comparison with the high doses of NO donor silica NPs reportedly required for the treatment of *S. epidermidis* biofilms [22], the sugar-mediated NO/THCPSi NPs showed effective biofilm reduction at a fractional dose.

### Cytotoxicity of NO/THCPSi NPs to NIH/3T3 fibroblast cells

**Figure 6:**
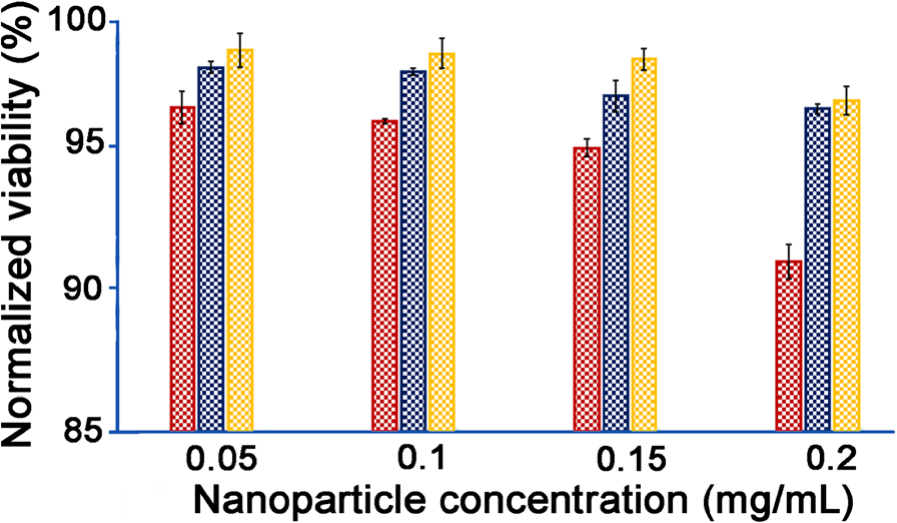
**Toxicity of the NPs to NIH/3T3 fibroblasts using the LDH assay after 48-h incubationc** NO/THCPSi NPs (red bars), glucose/THCPSi NPs (blue bars), and THCPSi NPs (yellow bars). Viability measures normalized to no NP control samples (*n* = 3; mean ± standard deviation shown).

The biocompatibility of THCPSi NPs has been previously reported by Santos and co-workers [25,28], where cytotoxicity, oxidative, and inflammatory responses were studied for a variety of mammalian cell lines. The toxicity of NO/THCPSi NPs, glucose/THCPSi NPs, and THCPSi NPs at different concentrations (0.05 to 0.2 mg/mL) over 48 h was evaluated using the NIH/3T3 cell line, which is one of the most commonly used fibroblast cell lines and often used as a model for skin cells. Two viability assays were used for toxicity studies: LDH and fluorescein diacetate-propidium iodide (FDA-PI). As shown in Figure 6, the results from the LDH assay showed well over 90% viability for all NP types up to 0.1 mg/mL. However, increasing the concentration of NO/THCPSi NPs to 0.2 mg/mL reduced the viability of NIH/3T3 cells to 92%. In contrast, the viability of fibroblast cells incubated with glucose/THCPSi NPs and THCPSi NPs at 0.15 and 0.2 mg/mL remained over 95%. The results of the FDA-PI assay (Additional file 1: Figure S3) were consistent with those obtained using the LDH assay.

The cytotoxicity of THCPSi NPs has been reported to be concentration dependent [25,27], and increased concentrations of NO/THCPSi NPs did raise cytotoxicity. However, the cytotoxicity of THCPSi NPs on fibroblast cells is much less than observed for silica NPs, silver NPs, and other clinical antiseptic wound treatments [3,11,44,45]. We note that dosage optimization (e.g., concentration of 0.1 mg/mL) enables a balance between high antibacterial efficacy and low toxicity towards mammalian cells present in a wound environment to be achieved.

## Conclusions

The present work demonstrates the capacity of THCPSi NPs to be loaded with NO by utilizing the sugar-mediated thermal reduction of nitrite. These NO/THCPSi NPs possess the capacity to deliver NO at therapeutic levels in a more sustained manner than previously demonstrated using NO-releasing NPs. NO delivered from the NPs was effective at killing pathogenic *P. aeruginosa*, *E. coli*, and *S. aureus* after only 2 h of incubation. After 24 h, the bacterial load was reduced by approximately 1 log. In addition, NO/THCPSi NPs showed effectiveness at inhibiting the growth of biofilm-based microbes. The NO/THCPSi NPs demonstrated a 47% reduction in *S. epidermidis* biofilm viability compared to the control samples. On the other hand, NIH/3T3 mouse fibroblasts incubated with the same concentration of NO/THCPSi NPs for 48 h maintained high cell viability. In summary, our results suggest that NO/THCPSi NPs are useful as a nanocarrier for NO release to treat bacterial infections in wounds. Future studies will focus on enhancing NO release and identifying the interactions between NO/THCPSi NPs and bacterial cell membranes.

## Abbreviations

CFU: colony-forming unit; DAF-FM: 4-amino-5-methylamino-2*'*,7*'*-difluorofluorescein; FDA: fluorescein diacetate; FBS: fetal bovine serum; glucose/THCPSi NPs: glucose-loaded thermal hydrocarbonized nanoparticles; LDH: lactate dehydrogenase; MRSA: methicillin-resistant *Staphylococcus aureus*; NO/THCPSi NPs: nitric oxide-loaded thermal hydrocarbonized nanoparticles; NP: nanoparticle; PI: propidium iodide; PSi: porous silicon; THC: thermal hydrocarbonized/hydrocarbonization; TSB: tryptic soy broth.

## Competing interests

The authors declare that they have no competing interests.

## Authors’ contributions

NHV, MHK, JS, and KV conceived and designed the experiments. MHK, AC, and BD performed the experiments. MHK, AC, FJH, BD, and NHV analyzed the data. MHK, AC, BD, FJH, SJPM, EM, JS, KV, and NHV wrote the paper. All authors read and approved the final manuscript.

## Supplementary Material

Additional file 1: Figure S1 Representative scanning electron microscope (SEM) image of THCPSi NPs (a) and DLS size distribution of THCPSi NPs (b). **Figure S2.** fluorescence detection of NO released from NO/THCPSi NPs. (a) Calibration curve obtained by adding aliquots of saturated NO solution (1.87 mM) to PBS containing DAF-FM indicator. (b) NO detection from NO/THCPSi NPs, glucose/THCPSi NPs (control), sodium nitrite/THCPSi NPs (control), sodium nitrite (control), and PBS (control) prepared using the heating protocol after 2 h of the release process at 37°C. **Figure S3.** cytotoxicity of (A) NO/THCPSi NPs, (B) glucose/THCPSi NPs, (C) THCPSi NPs, and (D) no treatment control towards NIH/3T3 cells as measured by FDA-PI assay after 48 h. The roman numbers represent the different concentrations of the NPs (I 0.05 mg/mL, II 0.1 mg/mL, III 0.15 mg/mL, and IV 0.2 mg/mL). Click here for file
