# Supplementary material for: Nitric oxide-releasing porous silicon nanoparticles
Source: Nanoscale Res Lett. 2014 Jul 4;9(1):333. doi: 10.1186/1556-276X-9-333 (PMC4109794; doi:10.1186/1556-276X-9-333)
Supplement: Additional file 1: Figure S1 — Representative scanning electron microscope (SEM) image of THCPSi NPs (a) and DLS size distribution of THCPSi NPs (b). Figure S2. fluorescence detection of NO released from NO/THCPSi NPs. (a) Calibration curve obtained by adding aliquots of saturated NO solution (1.87 mM) to PBS containing DAF-FM indicator. (b) NO detection from NO/THCPSi NPs, glucose/THCPSi NPs (control), sodium nitrite/THCPSi NPs (control), sodium nitrite (control), and PBS (control) prepared using the heating protocol after 2 h of the release process at 37°C. Figure S3. cytotoxicity of (A) NO/THCPSi NPs, (B) glucose/THCPSi NPs, (C) THCPSi NPs, and (D) no treatment control towards NIH/3T3 cells as measured by FDA-PI assay after 48 h. The roman numbers represent the different concentrations of the NPs (I 0.05 mg/mL, II 0.1 mg/mL, III 0.15 mg/mL, and IV 0.2 mg/mL). [file 1556-276X-9-333-S1.docx]

Additional file

**Nitric Oxide Releasing Porous Silicon Nanoparticles**

Morteza Hasanzadeh Kafshgari^1^, Alex Cavallaro^1^, Bahman Delalat^1^, Frances J. harding^1^, Steven J.P. McInnes^1^, Ermei Mäkilä^2^, Jarno Salonen^2^, Krasimir Vasilev^1^, Nicolas H. Voelcker^1,*^

^1^Mawson Institute, University of South Australia, Adelaide 5095, South Australia
^2^Department of Physics and Astronomy, University of Turku, FI-20014 Turku, Finland

**
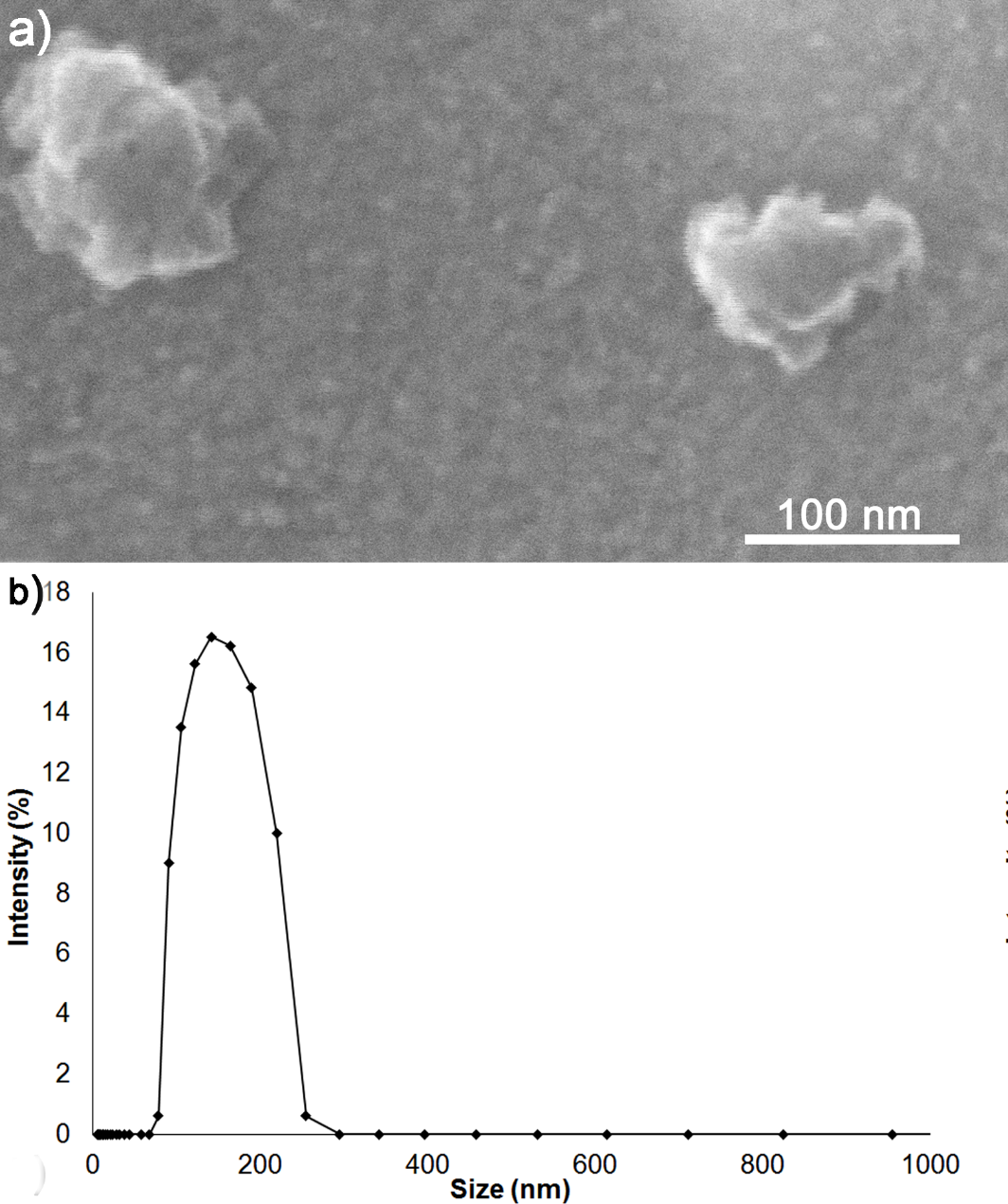
**

**Additional file 1: Figure S1.** Representative scanning electron microscope (SEM) image of THCpSiNPs (a) and DLS size distribution of THCpSiNPs (b).

**
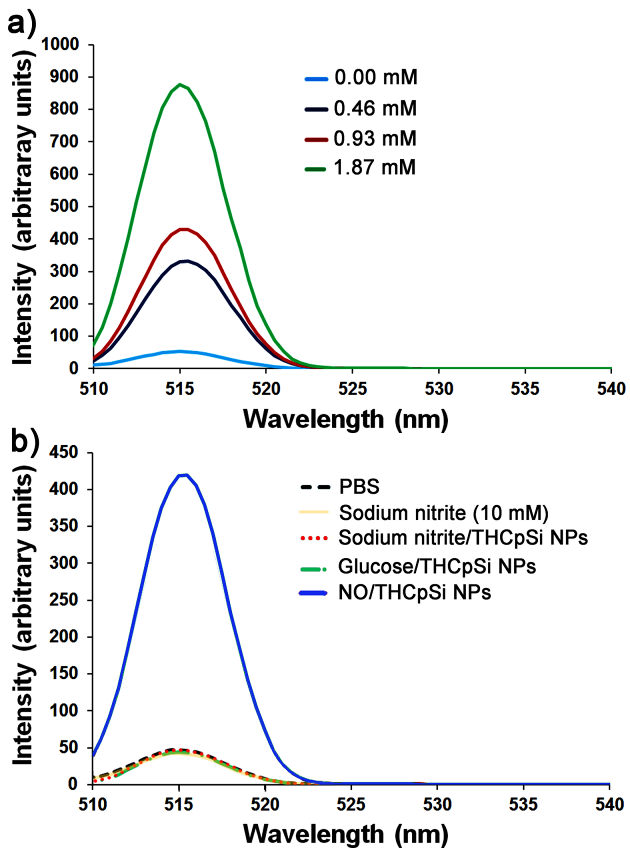
**

**Additional file 1: Figure S2.** Fluorescence detection of NO released from NO/THCpSiNPs; (a) Calibration curve obtained by adding aliquots of saturated NO solution (1.87 mM) to PBS containing DAF-FM indicator, (b) NO detection from NO/THCpSiNPs, glucose/THCpSiNPs (control), sodium nitrite/THCpSiNPs (control), sodium nitrite (control) and PBS (control) prepared using the heating protocol after 2 h of the release process at 37 ^o^C.

**
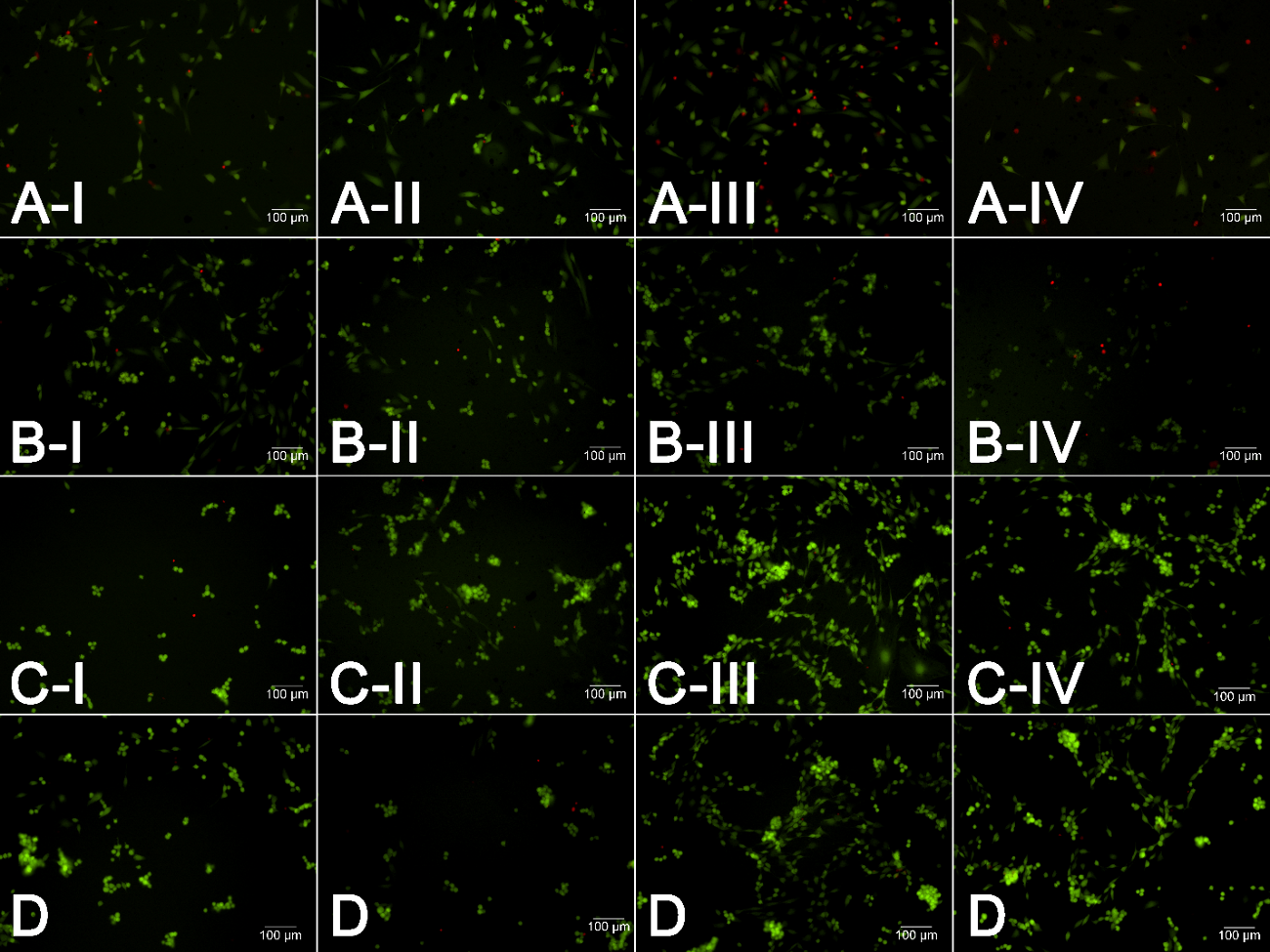
**

**Additional file 1: Figure S3.** Cytotoxicity of (A) NO/THCpSiNPs, (B) glucose/THCpSiNPs, (C) THCpSiNPs and (D) no treatment control towards NIH/3T3 cells as measured by FDA-PI assay after 48 h. The roman numbers represent the different concentrations of the NPs (I: 0.05 mg/mL, II: 0.1 mg/mL, III: 0.15 mg/mL, and IV: 0.2 mg/mL).
